# Comprehensive analysis of NMR data using advanced line shape fitting

**DOI:** 10.1007/s10858-017-0141-6

**Published:** 2017-10-17

**Authors:** Markus Niklasson, Renee Otten, Alexandra Ahlner, Cecilia Andresen, Judith Schlagnitweit, Katja Petzold, Patrik Lundström

**Affiliations:** 1 Division of Chemistry, Department of Physics, Chemistry and Biology, Linköping University, 58183 Linköping, Sweden; 2 Howard Hughes Medical Institute and Department of Biochemistry, Brandeis University, 415 South Street, Waltham, MA 02454 USA; 3 Department of Medical Biochemistry and Biophysics, Karolinska Institute, 17177 Stockholm, Sweden

**Keywords:** Peak integration, Line shape fitting, Spectral analysis, Relaxation, Dynamics

## Abstract

NMR spectroscopy is uniquely suited for atomic resolution studies of biomolecules such as proteins, nucleic acids and metabolites, since detailed information on structure and dynamics are encoded in positions and line shapes of peaks in NMR spectra. Unfortunately, accurate determination of these parameters is often complicated and time consuming, in part due to the need for different software at the various analysis steps and for validating the results. Here, we present an integrated, cross-platform and open-source software that is significantly more versatile than the typical line shape fitting application. The software is a completely redesigned version of PINT (https://pint-nmr.github.io/PINT/). It features a graphical user interface and includes functionality for peak picking, editing of peak lists and line shape fitting. In addition, the obtained peak intensities can be used directly to extract, for instance, relaxation rates, heteronuclear NOE values and exchange parameters. In contrast to most available software the entire process from spectral visualization to preparation of publication-ready figures is done solely using PINT and often within minutes, thereby, increasing productivity for users of all experience levels. Unique to the software are also the outstanding tools for evaluating the quality of the fitting results and extensive, but easy-to-use, customization of the fitting protocol and graphical output. In this communication, we describe the features of the new version of PINT and benchmark its performance.

**Electronic supplementary material:**

The online version of this article (doi:10.1007/s10858-017-0141-6) contains supplementary material, which is available to authorized users.

## Introduction

The last decades have brought dramatic improvements in methodology for measurement and analysis of the dynamics of proteins, nucleic acids and other biomolecules at multiple time scales. Motions on the picosecond to nanosecond time scale are analyzed by measurements of longitudinal and transverse relaxation rate constants and the heteronuclear NOE, and are usually interpreted according to the model-free formalism (Clore et al. 1990; Lipari and Szabo 1982a, b). This yields the diffusion tensor for molecular reorientation as well as amplitudes and time constants for the fluctuation of bond vectors. From the former it is possible to determine the effective size and anisotropy of the molecule (Bruschweiler et al. 1995), whereas the latter is a measure of configurational entropy (Akke et al. 1993). Motions that are slower than molecular tumbling manifest as an exchange contribution to the transverse relaxation rate constant that can be decomposed into exchange rate constants, populations of exchanging states and their difference in chemical shift. Depending on the time scale of the exchange process, Carr–Purcell–Meiboom–Gill (CPMG) relaxation dispersion (RD) (Allerhand and Thiele 1966; Carr and Purcell 1954; Meiboom and Gill 1958), longitudinal relaxation in the rotating frame (R_1ρ_) RD (Jones 1966), chemical exchange saturation transfer (CEST) (Forsén and Hoffman 1963) or ZZ-exchange (Jeener et al. 1979) experiments are performed and the data is fitted to the Bloch-McConnell equations or limiting cases thereof (McConnell 1958; Palmer et al. 2001). The methodology has given important insights into biological processes such as protein folding, enzymatic catalysis and ligand binding (Boehr et al. 2006; Korzhnev et al. 2004; Vallurupalli et al. 2008). Although most studies concern applications to proteins, the methodology has been extended to characterization of dynamics on various time scales for nucleic acids (Akke et al. 1997; Dethoff et al. 2012).

Central for the above achievements are accurate, robust and efficient methods for determining peak intensities in NMR spectra and for converting these into relaxation rates and other quantities. In spectra of complex biomolecules, the preferred method for determining peak intensities is line shape fitting (commonly referred to as integration) and several applications for this purpose exist (Delaglio et al. 1995; Hansen et al. 2007; Lee et al. 2015; Vranken et al. 2005). In 2013, we developed Peak INTegration (PINT) (Ahlner et al. 2013) that is used in a homologous fashion as similar software including the NMRPipe suite (Delaglio et al. 1995) and most notably FuDa (Hansen et al. 2007). While PINT performed well in terms of accuracy and speed, we identified a number of shortcomings that it shares with the aforementioned applications. Most importantly, it was not self-contained but relied on auxiliary software and scripts for tasks such as the modification of peak lists, validation of results and preparation of graphs. This and the lack of a graphical user interface (GUI) presented a significant obstacle, especially for novice users. Additional shortcomings were missing flexibility concerning format of spectral data and that the software was not available for the Windows operating system.

To address this, we have designed a completely new version of PINT where all tasks are integrated into a single application with an intuitive GUI. It is thus possible now to perform the entire analysis from spectral visualization and peak lists optimization to the preparation of high-quality plots with relaxation parameters in PINT. The GUI facilitates to gauge the quality of the fits as well as to diagnose and address the cause of unsatisfactory results. Furthermore, the use of multithreading and further optimization of the algorithms resulted in a significant speed-up of the line shape fitting and additional downstream fitting options are included that increase the versatility of the program. To reduce the time required for learning how to use PINT, several tutorial videos, an extensive help section and various example projects are bundled with the open-source software that is available for Windows, macOS and Linux. The video in Online Resource 1 is a short trailer for PINT that gives a brief introduction to the appearance and features of the software. In the following we will describe the new features of PINT and how it leads to improved productivity.

## Results and discussion

### Usage

**Fig. 1:**
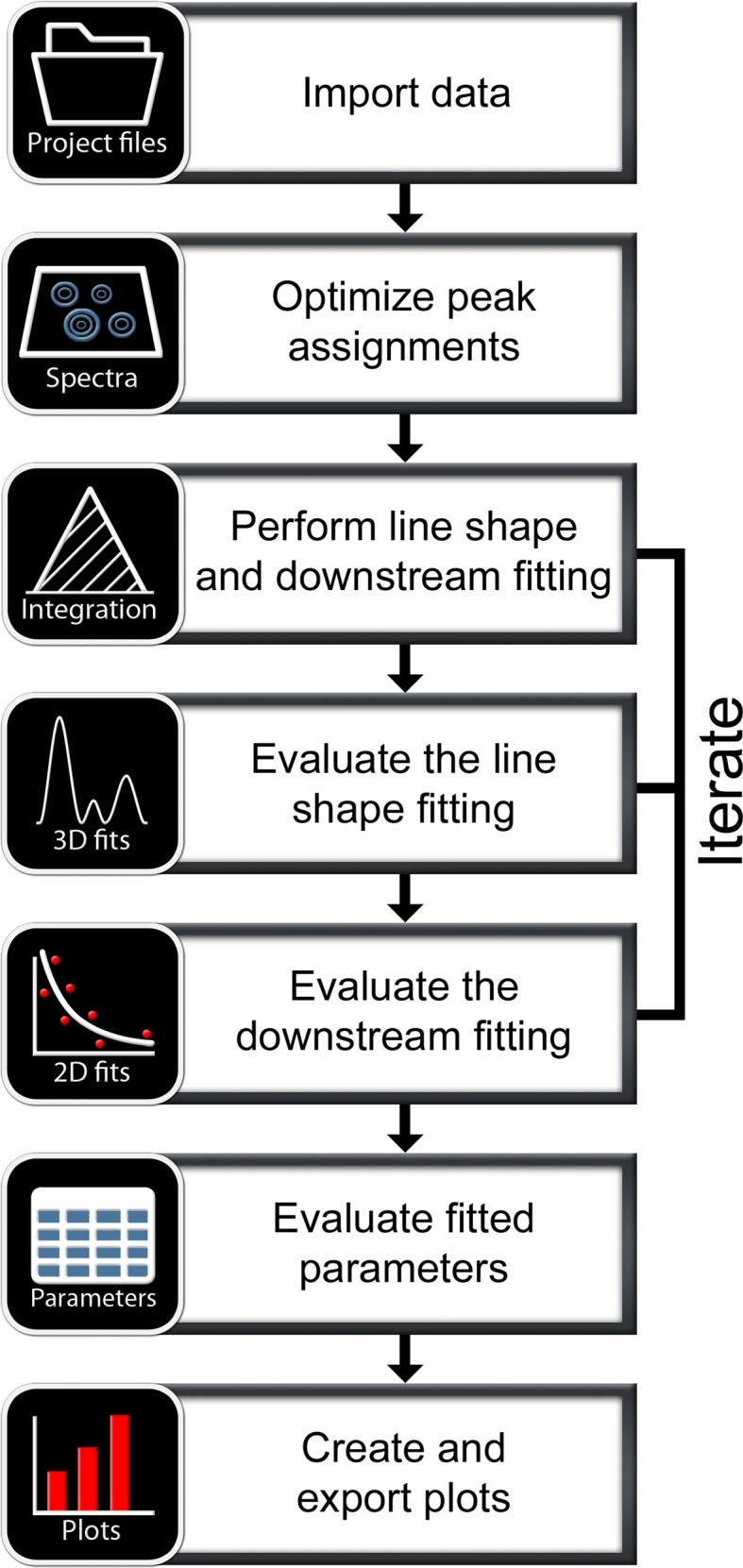
Organization and proposed workflow in PINT. The functionality of PINT is subdivided into seven tabs. Each tab has a specific purpose, which makes PINT intuitive to use while freeing up the user interface from unnecessary options. It is always possible to review a previous tab and whenever there are new results available for evaluation, this is clearly indicated in the appropriate tabs

The GUI of the main workspace in PINT consists of seven tabs corresponding to the typical workflow of line shape fitting and downstream analysis of the obtained peak intensities (Fig. 1). The different tabs let the user import spectra, peak lists and other data; view and interact with spectra; perform line shape fitting and fitting of obtained intensities; analyze the quality of the line shape fitting, inspect decay rates and similar profiles; and, finally, to plot relaxation rate constants and other parameters. Since analysis of NMR data frequently is an iterative process, it is often necessary to return to a previous step and modify the parameters.

**Fig. 2:**
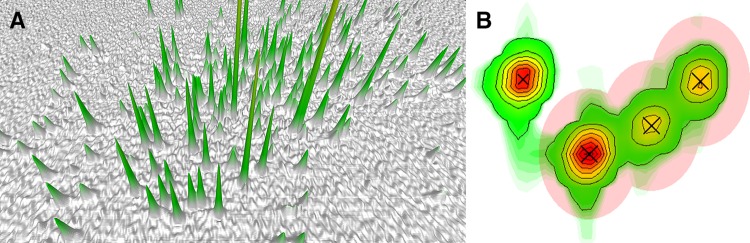
The different modes for spectral visualization. **a** 3D representation of a two-dimensional NMR spectrum. Due to the quality of the 3D graphics, it is often simple to distinguish peaks from noise and to detect overlap using this mode. **b** Section of a two-dimensional spectrum represented as a contour plot and spectrogram. Peaks are indicated by crosses and for clarity assignments are not shown. In the example, a group of three partially overlapped peaks that must be fitted together is shown. The areas for integration (translucent red) have been adjusted to exclude empty regions and nearby peaks that can be integrated separately. This adjustment is done graphically in the spectral viewer

The functionality of the spectral viewer is reminiscent of similar applications and should feel familiar to experienced users. PINT supports two-dimensional and pseudo three-dimensional spectra processed in NMRPipe (Delaglio et al. 1995) or TopSpin™ (Bruker Corporation). The spectra can be visualized as contour plots with or without spectrograms or using a state-of-the-art 3D viewer. While the contour plot representation usually provides a better overview, the latter mode is useful for discriminating between true peaks and noise as well as for detecting overlaps. The excellent quality of the spectral viewer is shown in Fig. 2 and dedicated tools detailed in the help section and the instructional videos facilitate efficient spectral interaction. If a pseudo three-dimensional spectrum or several two-dimensional spectra have been imported, it is straightforward to browse the different planes or spectra.

Peak assignments can be imported from any text file and are shown in the spectrum and a synchronized table for easy navigation. Existing peaks can be repositioned, folded, deleted or re-assigned and peak picking of new cross peaks is supported. PINT features two different routines for peak centering, one that can handle overlapped peaks provided that they are close to their correct positions and one that works well for isolated peaks further from peak center. If a peak list is unavailable, all peaks with intensities above a chosen threshold can be automatically picked and given dummy assignments according to a customizable format.

The main purpose of the spectral viewer is to aid the user in selecting the appropriate regions for line shape fitting of the various peaks and determining whether peaks must be fitted together as a group because of spectral overlap. Figure 2b shows a group of three such peaks and some peripheral peaks that can be excluded from the group. In the spectral viewer, one can conveniently define overlapped groups and adjust the area considered for line shape fitting globally or for selected peaks.

All PINT requires for basic line shape fitting is one or several spectra and a common peak list, but there are several ways to customize the line shape fitting if necessary or desired. For instance, the default Gaussian line shape can be changed to Lorentzian, Galore (linear superposition of Gaussian and Lorentzian line shapes of equal line widths) or Voigt (convolution of Gaussian and Lorentzian line shapes of unequal line widths); overlaps and areas for integration can be entered or reviewed manually; and initial estimates of parameters can be modified. It is also possible to fix peak positions and line widths to certain values rather than to fit them. In fact, PINT includes on the order of one hundred options, most of which can be applied globally or to selected peaks, that are described in the help section and the instructional videos. The options are entered in a text browser with autocomplete functionality to improve speed and to avoid typing mistakes. Furthermore, commonly used parameter sets can be saved as templates and loaded into future projects to avoid entering options manually. A default template that is automatically loaded for all newly created projects can also be defined.

**Fig. 3:**
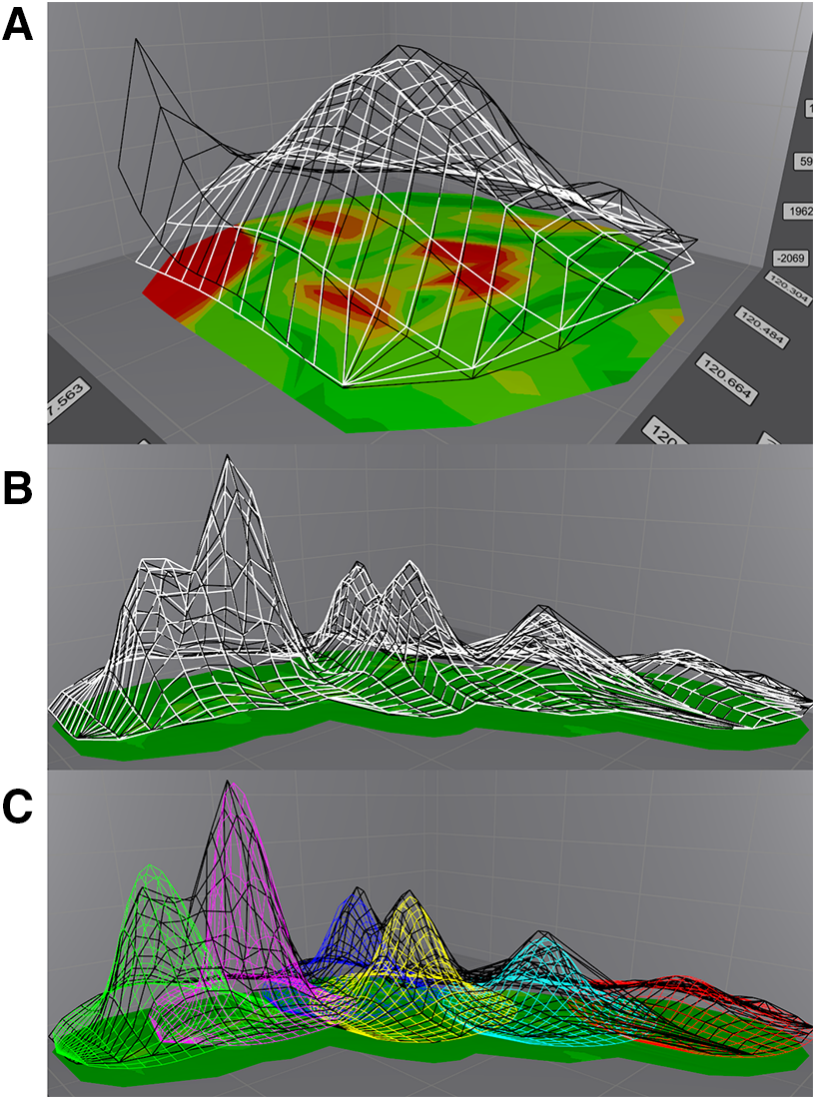
Inspecting the results from the line shape fitting. Experimental (black lines) and fitted (colored lines) data are shown as superimposed mesh plots on top of a color-coded difference map where green indicates good agreement. **a** A poorly fitted peak manifests as visible deviations between experimental and fitted data, resulting in yellow and red regions in the difference map. In this case, the reason for the poor fit is unconsidered overlaps. **b** The result when the appropriate overlaps are considered. Here, the superposition of fitted data for all peaks that are integrated together are displayed. It is also possible to visualize fits for the various peaks individually in different colors as shown in **c**

One of the most obvious advantages of PINT compared to other software is the ease by which the quality of the line shape fitting can be checked and addressed. The tab “3D fits” features plots containing superimposed experimental and fitted line shapes on top of a color-coded difference map that clearly signals issues encountered during integration. Figure 3 shows examples of poor and satisfactory fits of line shapes. If a peak has not been integrated to satisfaction a shortcut can be used to open the spectral viewer and zoom in on the region of interest where the cause can be identified. Areas considered for integration and definitions of overlaps can then be swiftly modified and used in a subsequent integration. An annotated example of a customized fit is shown in Online Resource 2. Since some groups of peaks can require extensive optimization, PINT includes an option to only fit a selected subset of peaks.

The typical purpose of line shape fitting is to determine peak intensities as a function of an experimental parameter and then propagate these into, for instance, relaxation rate constants or parameters related to conformational dynamics. PINT includes ten expressions for downstream fitting of peak intensities (Online Resource 2). These include multipurpose functions such as the linear function and the exponential function. There are also dedicated functions for fitting Carr–Purcell–Meiboom–Gill (CPMG) relaxation dispersions to the corrected Carver–Richards equation (Carver and Richards 1972; Davis et al. 1994) and for fitting saturation recovery or inversion recovery data. In addition, the heteronuclear NOE can be calculated from ratios of peak intensities and R_1ρ_ relaxation rate constants (together with R_1_ rate constants if available) can be converted to R_2_ relaxation rate constants (Jones 1966). Uncertainties in the model parameters are estimated by jackknife or bootstrap resampling or by Monte Carlo simulations (Efron and Tibshirani 1993; Mosteller and Tukey 1977; Press et al. 1988).

**Fig. 4:**
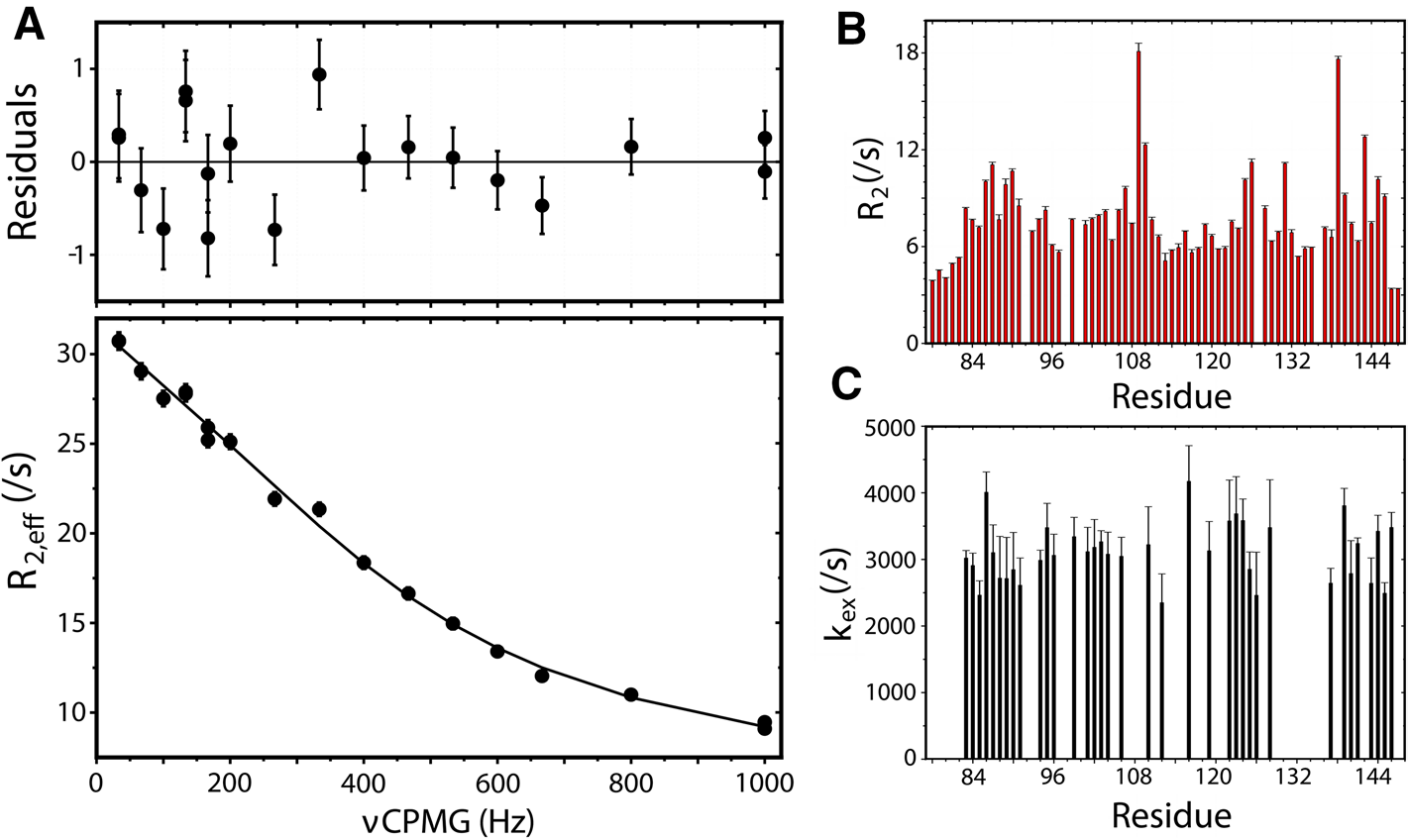
PINT produces graphs that can be exported and directly used with vector based software. The panels of this figure represent analysis of data acquired for calcium-free calmodulin C-terminal domain that have been exported as .pdf files and enhanced (fonts and thickness of lines) in Adobe^®^ Illustrator prior publication. The figure shows a subset of different types of plots that can be generated in PINT. **a** 15N CPMG relaxation dispersion profile for residue K94 fitted to the Carver–Richards equation. The residuals are shown above the dispersion profile. **b** Summary of transverse relaxation rate constants determined from R1ρ and R1 relaxation experiments. Similar plots can be prepared for any determined parameter. **c** Summary of exchange rate constants determined from 15N CPMG relaxation dispersion experiments. Data is shown for residues with significant (p < 0.01) exchange as established by F-tests performed in PINT

From plots of fitted curves and peak intensities, it is easy to gauge if the fits are satisfactory and the adjacent plots of the residuals are useful for detecting systematic bias. In case of unsatisfactory results, it is possible to customize initial estimates of the model parameters or, in the case of CPMG relaxation dispersions, perform a grid search prior to refitting. To save time, this can be done without reintegrating the peaks. All graphs can be exported as images in .png or .pdf format. Files of the latter format are vector based and can be modified in, for instance, Adobe^®^ Illustrator to yield publication quality figures. Figure 4a shows the ^15^N CPMG relaxation dispersion profile for K94 of calcium-free C-terminal domain of calmodulin that has been prepared by this method.

The final step in PINT is to summarize and present results for all peaks. In the tab “Parameters” all parameters from the line shape fitting and downstream analysis are shown. These include optimized peak positions and line widths, intensities and volumes as well as all determined parameters from fits of intensities. For most parameters, their associated uncertainties are also shown. Any of parameters can be plotted as a function of residue number within PINT in the formats described above for all or selected peaks. This is shown in Fig. 4 where transverse relaxation rate constants determined from R_1ρ_ and R_1_ experiments for all peaks are presented in panel B and exchange rate constants determined from CPMG relaxation dispersions experiments for peaks with significant exchange are shown in panel C. All parameters are automatically saved as text files for processing or plotting in external applications if desired.

### Performance

Compared to the previous version of PINT, integration speed has improved by an order of magnitude. This has been accomplished by optimization of the algorithms and taking advantage of multithreading. The GUI, with the possibility to more easily optimize peak lists and define overlaps and areas for integration appropriately, leads to considerable further time savings. To benchmark PINT, we used a computer equipped with a quad-core i7-4790 CPU @ 3.60 GHz running eight threads simultaneously and used data from various experiments for three different proteins.

For an SH3 domain of Abp1p (7 kDa, 62 residues) (Drubin et al. 1990; Lila and Drubin 1997; Rath and Davidson 2000), a small protein with a well-resolved HSQC spectrum, successful integration and fitting of R_1_ (20 relaxation delays), R_1ρ_ (17 relaxation delays) and CPMG relaxation dispersion (17 effective fields) data was complete in 2, 2 and 3 s, respectively. For these three examples, line shape fitting was performed using default parameters (Gaussian line shapes) and automatically detected overlaps. Our second example protein is calcium-free C-terminal domain of calmodulin (8.4 kDa, 73 residues) (Cheung 1970; Kakiuchi and Yamazaki 1970; Walsh et al. 1977). Although this protein is of the similar size as Abp1p SH3 domain, the HSQC features extensive overlaps and the largest overlapped group comprises eleven peaks compared to three for Abp1p SH3 domain. In this case, the times required for integration and fitting of R_1_ (25 relaxation delays), R_1ρ_ (20 relaxation delays) and CPMG relaxation dispersion (20 effective fields) data using default parameters were 29, 39 and 154 s, respectively. Furthermore, the line shape fitting did not converge in 1000 iterations for one group of seven peaks. When the line shape for these problematic peaks was changed to Galore, fitting was successful for all peaks and the required times were unchanged for R_1_ and R_1ρ_ and down to 30 s for CPMG relaxation dispersion. This time can be further reduced significantly by reviewing the areas considered for integration without compromising the quality of the line shape fitting. For example, using Gaussian (58 peaks) and Galore (13 peaks) line shapes and decreasing integration radius in the indirect dimension to 0.4 ppm instead of using the default value of 0.6 ppm, the CPMG relaxation dispersion data was fitted in 14 s. Our last example protein, the kinase domain of EphB2 (32 kDa, 285 residues), represents a large protein with a complex HSQC spectrum (Pasquale 1991; Wiesner et al. 2006). In addition to R_1_ (26 relaxation delays) and CPMG relaxation dispersion (19 effective fields) experiments, data from a chemical exchange saturation transfer CEST experiment (70 offsets) was integrated. The required times for integration and downstream fitting using Gaussian line shapes and custom areas for integration in this case were 20, 25 and 138 s, respectively. It is thus feasible to analyze even very large data sets within a reasonable time frame.

### Concluding remarks

We have developed an integrated open-source, cross-platform application for line shape fitting and determination of relaxation rate constants and quantification of other parameters. The GUI, the integrated spectral viewer and the potential to promptly identify and address issues using the custom options will lead to increased throughput. Users will also benefit from the straightforward generation of high quality images. Owing to the intuitive design, bundled example projects, a detailed help section and instructional videos, the software is accessible for users of all experience levels without compromising accuracy and versatility. Since the software is open-source, advanced users can also modify or add functionality. It is for instance straightforward to implement equations for additional line shapes and expressions to fit peak intensities to. One can also envisage more ambitious extensions such as co-fitting of data acquired at multiple static magnetic fields and model-free analysis of relaxation data. In summary, PINT has the potential to be a valuable tool for analysis of two-dimensional NMR spectral data including applications beyond the intended scope.

## Distributed files

Binaries for 64-bit versions of Windows, macOS and Linux as well as source code can be downloaded at https://pint-nmr.github.io/PINT/. PINT is written in C++ and is an open-source software released under the terms of the GNU General Public License (http://www.gnu.org/licenses/). PINT was compiled in Qt Creator 3.6.83 using libraries from Qt 5.7 open source license (https://www.qt.io/download-open-source/#section-2), Qwt (http://qwt.sourceforge.net/) and QCustomPlot (http://www.qcustomplot.com/). The Faddeeva Package (http://ab-initio.mit.edu/wiki/index.php/Faddeeva_Package) was used to write routines for fitting of Voigt line shapes and the Geometric Tools Engine (https://www.geometrictools.com) was used to detect overlapping peaks.

## Electronic supplementary material

Below is the link to the electronic supplementary material.

Supplementary material 1 (DOCX 131 KB)
