# Supplementary material for: Comprehensive analysis of NMR data using advanced line shape fitting
Source: J Biomol NMR. 2017 Oct 17;69(2):93–9. doi: 10.1007/s10858-017-0141-6 (PMC5662661; doi:10.1007/s10858-017-0141-6)
Supplement: Supplementary file 1 — Supplementary material 1 (DOCX 131 KB) [file 10858_2017_141_MOESM1_ESM.docx]

# Comprehensive analysis of NMR data using advanced line shape fitting

Supplementary material

Markus Niklasson^1,^*, Renee Otten^2^, Alexandra Ahlner^1,†^, Cecilia Andresen^1,†^, Judith Schlagnitweit^3,†^, Katja Petzold^3^ and Patrik Lundström^1,^*

^1^Division of Chemistry, Department of Physics, Chemistry and Biology, Linköping University, SE-58183 Linköping, Sweden

^2^Howard Hughes Medical Institute and Dept. of Biochemistry, Brandeis University, 415 South Street, Waltham, MA 02454, USA

^3^Dept. of Medical Biochemistry and Biophysics, Karolinska Institute, SE-17177 Stockholm, Sweden

^†^These authors contributed equally

*To whom correspondence should be addressed:

marni@ifm.liu.se (MN), patlu@ifm.liu.se (PL)

Keywords:

peak integration, line shape fitting, spectral analysis, relaxation, dynamics


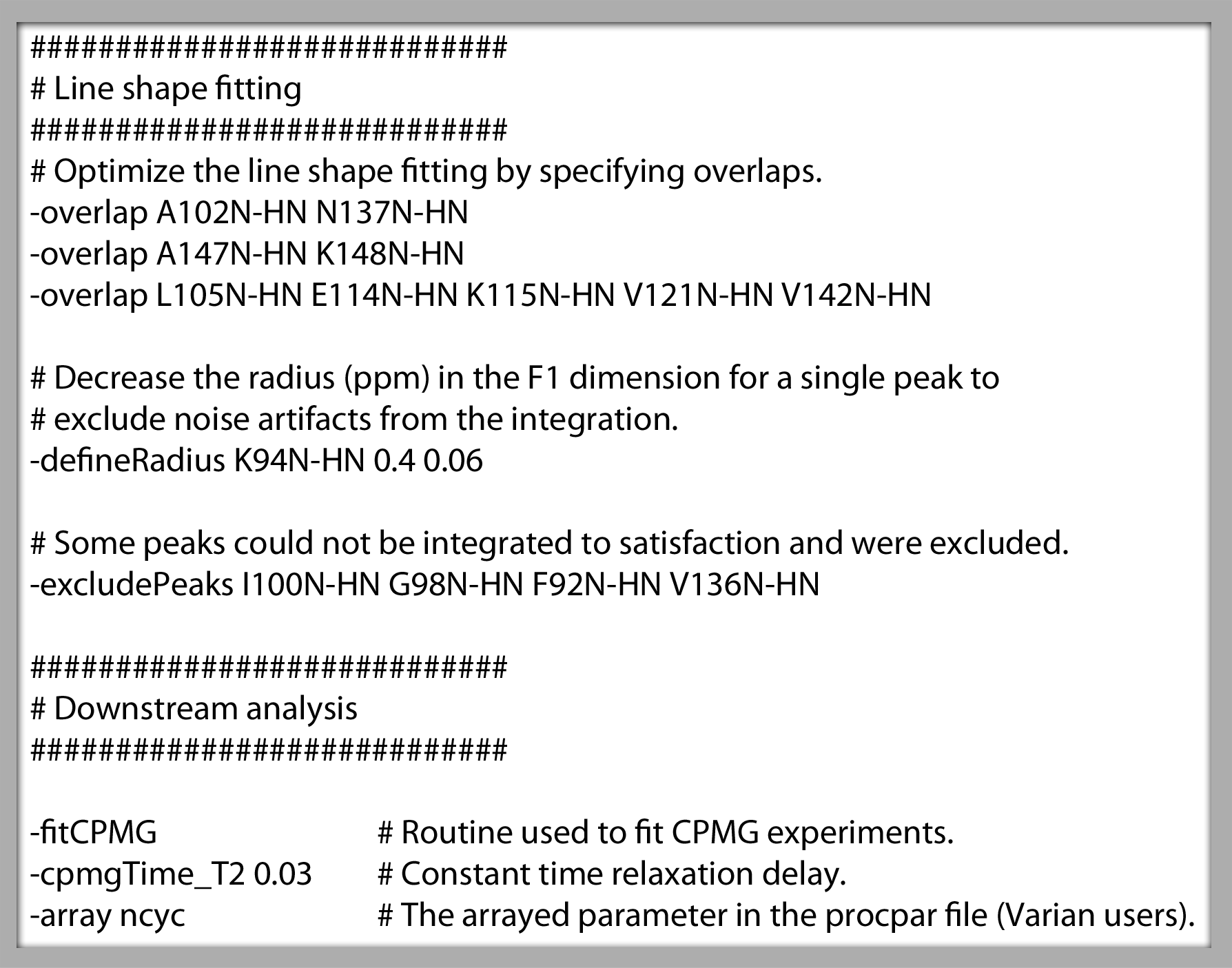


**Supplementary Fig. 1** Example of customized peak integration and analysis of ^15^N CPMG relaxation dispersions. Simple one line statements are written in a text browser with autocomplete functionality. In this example, overlapped peaks to fit together have been defined, the area considered for integration for one peak has been modified, peaks have been excluded from the analysis and options specific for the analysis of CPMG relaxation dispersion have been added.

**Supplementary Table 1** The fitting routines available in PINT.

| Option | Description | Equation |
| --- | --- | --- |
| fitConstant | Fits data to a constant function. | $y(x)=a$ |
| fitLin | Fits data to a linear function. | $y=ax+b$ |
| fitExp | Fits peak volumes to a decaying exponential function of a relaxation delay. | $y\left( x \right)=a\cdot\exp(-bx)$ |
| fitExpOffset | Fits peak volumes to a decaying exponential function with an offset of a relaxation delay. | $y\left( x \right)=a\cdot\exp\left( -bx \right)+c$ |
| fitBiExp | Fits a biexponential function and outputs volumes as a function of a relaxation delay. | $y\left( x \right)=a\cdot\exp\left( -bx \right)+c\cdot\exp\left( -dx \right)$ |
| fitInvRecovery | Fits inversion recovery data. | $y=a(1-b\exp(-cx))$ |
| fitSatRecovery | Fits saturation recovery data. | $y=a(1-\exp(-bx))$ |
| fitCPMG | Converts peak volumes to R_2,eff_(ν_CPMG_) and fits these to the Carver-Richards equations for two-site exchange. | Corrected Carver-Richards equations |
| fitR2fromR1rho | Fits R_1ρ_ rates and uses these together with B_1_, carrier and a list of R_1_ values (optional) to calculate R_2_. | First, $y\left( x \right)=a\cdot\exp(-R_{1\rho}x)$ and optionally $y\left( x \right)=\exp(-R_{1}x)$  then $R_{2}={R_{1\rho}}/{\sin^{2} \theta-{R_{1}}/{\tan^{2} \theta}}$ |
| calcNOE | Calculates heteronuclear NOE from the ratio of intensities in every other plane. | $y\left( x_{1},x_{2} \right)={x_{1}}/{x_{2}}$ |
